# Novel nonmagnetic abutment designs for facial prostheses: an experimental study

**DOI:** 10.55730/1300-0144.5953

**Published:** 2024-10-14

**Authors:** İrem SÖZEN YANIK, Ufuk ADALI, Jamila YASSINE, Franziska SCHMIDT, Wolfgang HANNAK, Bahadır ERSU

**Affiliations:** 1 Department of Prosthodontics, Faculty of Dentistry, Hacettepe University, Ankara, Turkiye; 2 Department of Prosthodontics, Geriatric Dentistry and Craniomandibular Disorders, Faculty of Dentistry, Charité University, Berlin, Germany

**Keywords:** Nonmagnetic abutment, magnetic abutment, implant, facial prostheses, magnetic resonance imaging

## Abstract

**Background/aim:**

This in vitro study was undertaken with the aim of evaluating and comparing the retentive forces of novel nonmagnetic abutment designs developed as alternatives to conventional magnetic abutments for facial prostheses.

**Materials and methods:**

A plexiglass model was constructed and two extraoral implants were placed in these blocks in a parallel position. Nonmagnetic abutments made of titanium were fabricated and screwed onto the implants. The nonmagnetic systems represent a novel design and include two different abutment designs (type 1 and type 2) with silicone attachments. Retentive force values for the three abutment types of a conventional magnetic system (CMS), the nonmagnetic abutment type 1 system (NMS1), and the nonmagnetic abutment type 2 system (NMS2) were measured at the 0th, 120th, 360th, 720th, and 1440th dislodging cycles using a test machine. Given the data’s distribution characteristics, nonparametric tests were used for analysis. The Kruskal–Wallis test was used to evaluate significant differences among groups, followed by Dunn’s posthoc test for specific group comparisons. The Friedman test compared the number of dislodging cycles for each group, and the Benjamini–Hochberg adjusted Wilcoxon sign-rank test was used for pairwise comparisons.

**Results:**

Both NMS1 and NMS2 exhibited significantly higher retentive forces compared to CMS for the same dislodging cycles (p < 0.01). The NMS1 group showed the highest initial retentive force (9.98 ± 0.89 N), followed by the NMS2 group (9.65 ± 0.35 N), but this difference was not statistically significant. Significant differences in retention force values were observed among the three groups across the dislodging cycles (p < 0.001). The lowest retentive force in the last dislodging cycle was observed in the CMS group (3.39 ± 0.04 N). Additionally, the retention forces decreased in all groups with each increasing dislodging cycle.

**Conclusion:**

The two newly developed nonmagnetic systems displayed higher retentive forces compared to the magnetic systems and can be considered viable alternative abutment options for facial prostheses.

## 1. Introduction

Facial prostheses are used for the reconstruction of facial defects [1]. Defects in these regions can be congenital, including malformation and developmental disturbances, or acquired and associated with pathologies such as necrotizing diseases, oncologic surgery, or trauma [2,3]. Prosthetic facial rehabilitation is effective, minimally invasive as no additional surgical procedure is required, cosmetically satisfying, and leads patients to an early social reintroduction [4,5]. Facial prostheses are generally made of silicone elastomers. In current practice, osseointegrated implants are commonly used for retaining and supporting these facial prostheses, and they are collectively named implant-supported prostheses [6–8]. After osseointegrated implants are successfully placed, a retaining system is necessary to attach the prostheses to the implant [9]. A retaining system includes an abutment (torqued onto the implant) and an attachment (located in the prostheses). Basic retaining systems include bar-clip, ball/o-ring, and magnet systems. The literature reports magnet systems to be the most commonly adopted retaining system, showing satisfactory results in retaining facial prostheses [10–12]. However, it should be noted that magnetic systems have a major drawback when magnetic resonance imaging (MRI) is required for any reason [11,13]. These ferromagnetic abutments lead to artifacts in MRI and are even contraindicated during imaging, requiring removal by the clinician before MRI and reinsertion after imaging [13,14]. This is especially troublesome for oncological patients with maxillofacial defects, as repeated MRI examinations may be essential for staging, treatment planning, and follow-up [15]. Additionally, in dentistry, magnets can cause tissue damage through two primary mechanisms. First, the constant magnetism surrounding the tissues can lead to physical effects. The second mechanism is through the chemical effects of alloys and corrosion products [16]. Two further disadvantages of magnetic retention are its low resistance to lateral forces, as the magnets merely slide across the faces of the keepers, and the degradation of the magnetic forces over time [17]. Furthermore, although bar retainers, another type of nonmagnetic attachment, exhibit higher retentive forces, studies have identified various issues related to implant-supported extraoral prostheses, including fractured bars, the need for clip activation, loosening of bar screws and abutments, substructure fractures, and loss of attachment between the silicone and the substructure [18–20].

Based on these considerations, the aims behind the present study were to design two novel imaging-compatible implant-supported nonmagnetic abutment systems (NMS1 and NMS2) for facial prostheses and compare the retention levels of these two different designs with conventional magnetic abutment systems (CMS) under in vitro conditions. The null hypothesis was that there would be no significant differences in the retention forces between the nonmagnetic and conventional magnetic abutments.

## 2. Materials and methods

### 2.1. Sample size

The power of the sample size was calculated using G*Power v3.1 (Heinrich-Heine University, Düsseldorf, Germany). A repeated measures analysis of variance (within–between interaction) power analysis was employed for three groups (abutment types) and five measurements (dislodging cycles). The analysis was conducted with a 5% type-I error, 80% power, and 0.25 effect size, resulting in a minimum sample size of nine per group. Considering the possibility of data loss, it was decided to include 10 samples in each group.

### 2.2. Experimental models

Plexiglass was chosen for its transparency, dimensional stability, and cost-effectiveness. Rectangular plexiglass blocks sized 40 × 20 × 20 mm and 40 × 20 × 35 mm were used to mimic the prosthesis and the facial bone of the patient, respectively. Those blocks were named P (prosthesis) and FB (facial bone), and identical sets were created for each group.

**Figure 1:**
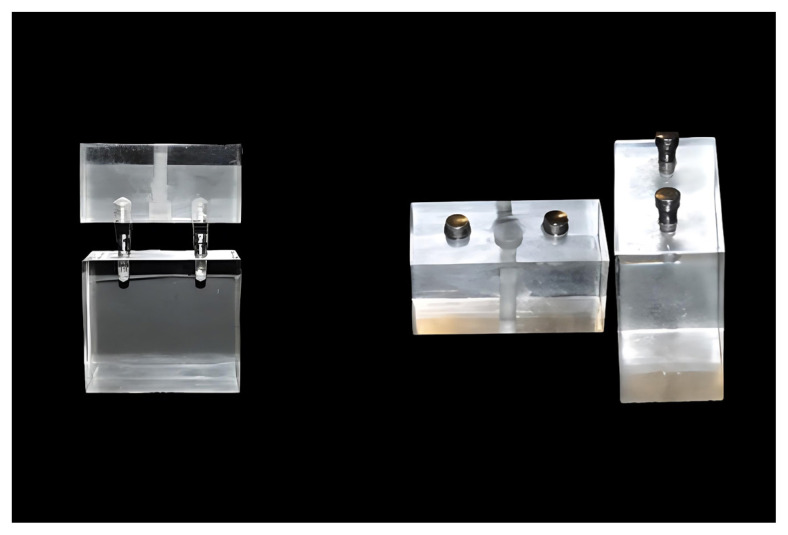
In vitro magnetic system model.

Two cone-type extraoral implants (EO implant ø 3.3 mm, 5 mm length; Institute Straumann AG, Switzerland) were inserted 20 mm apart and at identical depths into the FBs block of all three groups. These extraoral implants were placed parallel to each other at an interimplant distance of 20 mm using a parallelometer (Paraflex Parallelometer; BEGO, Bremen, Germany). For the CMS, the magnetic abutments were affixed to the two extraoral implants on the FB block using a torque of 15 newtons (N). Facing these abutments in the FB block, two housings were inserted into the P block, and magnetic attachments were placed inside these housings. The in vitro CMS model is shown in Figure 1.

**Figure 2:**
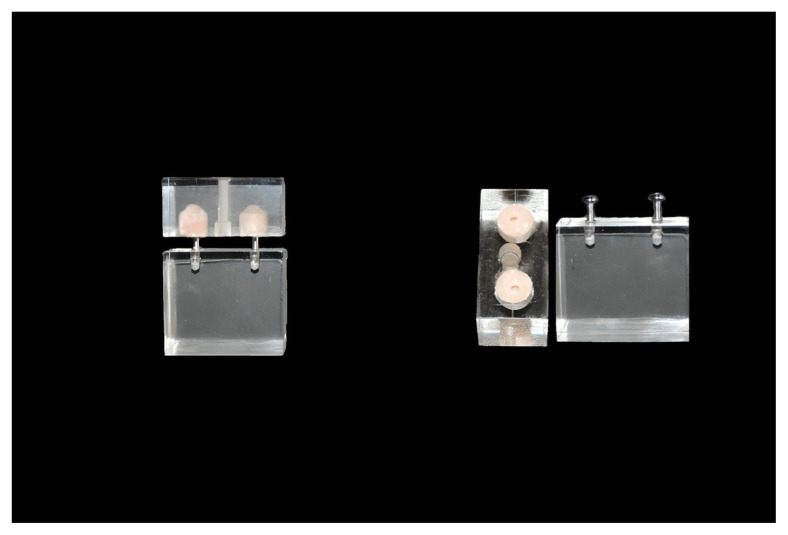
In vitro nonmagnetic system model.

For NMS1 and NMS2, two holes (10 mm in depth and 10 mm in diameter) were created inside the P block, facing the extraoral implants in the FB block. A silicone admixture (M511-Addition (Platinum) Silicone Rubber, 10:1 Technovent, Leeds, UK*)* was prepared according to the manufacturer’s instructions and used to fill the holes in the P block. Two nonmagnetic abutments were screwed into the extraoral implants on the FB block using a torque of 15 N. The silicone-filled P block and the abutment-placed FB block were aligned and fixed with a guide lock to ensure standardization. This was followed by a polymerization process at 100 °C for 1 h. The in vitro NMS model is shown in Figure 2.

### 2.3. Abutment systems

#### 2.3.1 Magnetic abutment

**Figure 3:**
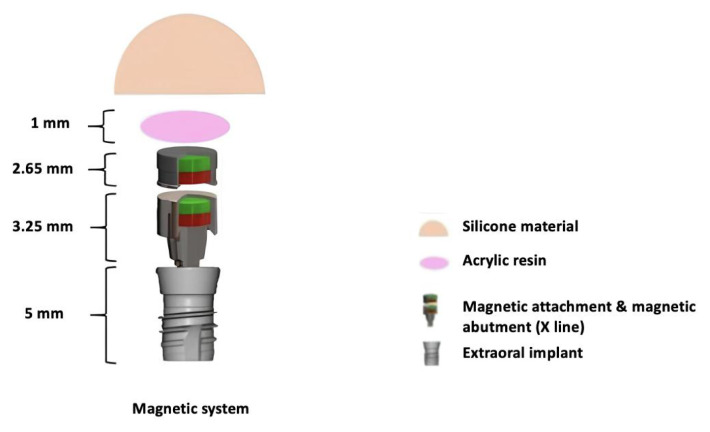
The components of the Steco Titanmagnetics X-Line magnetic system. The abutment is affixed to the extraoral implant, and the attachment is placed into the silicone prosthesis with the aid of acrylic resin.

Magnetic systems offer the advantage of self-alignment, which is critical for ensuring proper positioning and stability of prosthetic components. This self-aligning capability minimizes the need for manual adjustment and reduces the risk of misalignment. In this study, Steco Titanmagnetics X-Line (Steco-system-technik, Hamburg, Germany) was used as the magnetic abutment and attachment system, as shown in Figure 3. The magnets are made of samarium cobalt, which is more heat-resistant than neodymium magnets and can withstand sterilization. The Steco Titanmagnetics X-Line has a head diameter of 4.8 mm. It features magnet attachments that are 2.65 mm in height and magnet abutments with a length of 3.25 mm, ensuring secure and stable prosthetic connections.

#### 2.3.2 Nonmagnetic abutments

**Figure 4:**
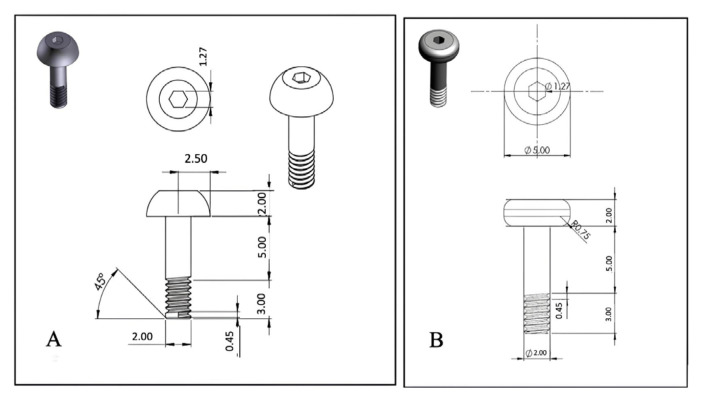
Diagrams of the nonmagnetic abutments. The head has a diameter of 5 mm and a height of 2 mm. The Allen’s head has a diameter of 1.27 mm. The thread is 3 mm long with a thread angle of 45°. (A) The underside of the type 1 abutment head is not rounded to provide maximum retention. (B) The underside of the type 2 abutment head is rounded to protect the silicone attachment.

The nonmagnetic abutment designs were created as three-dimensional (3D) computer-aided design models using SolidWorks, from which technical drawings were generated. The abutments were manufactured from Grade 5 titanium (Ti-6Al-4V) using a computer numerical control machine (HANWHA CNC Swiss Turning Lathe XP16-S; Gyeonggi-do, Korea). During the development of type 1 abutment, the primary goal was to ensure maximum retention, defined as resistance to dislodging forces. The retention of the type 1 abutment is provided by the wide lower part of the abutment head, which has not been rounded. In contrast, the design of the type 2 abutment includes a rounded lower edge of the abutment head to prevent damage to the silicone. The type 1 and type 2 abutments were designed as shown in Figures 4a and 4b. Dimensions for both nonmagnetic abutments are as follows:

- Thread angle: 45°
- Thread length: 3 mm
- Abutment head diameter: 5 mm
- Abutment head height: 2 mm
- Allen hole: 1.27 mm

**Figure 5:**
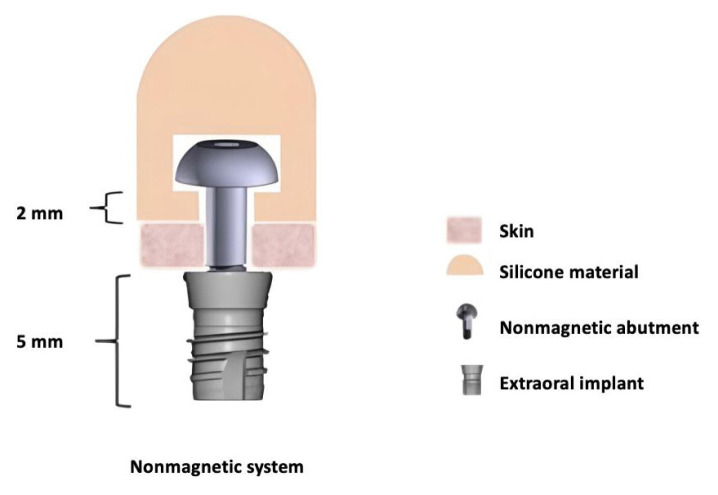
The components of the nonmagnetic systems. The abutment is screwed into the extraoral implant, and there is no attachment component in this system. The head of the abutment is directly inserted into the silicone prosthesis.

It was observed during this study that silicone prostheses tended to deform when the silicone thickness under the abutment head was less than 2 mm. Therefore, the unthreaded part of the abutment shaft needed to be longer than 2 mm. However, considering skin thickness, a length of 5 mm was preferred, as shown in Figure 5, so abutments of this size were produced for the two nonmagnetic abutment experimental groups.

### 2.4. Experimental groups

In this study, three experimental groups, each comprising 10 samples, were evaluated to assess the performance of various abutment systems in prosthetic applications. The groups were defined as follows:

- Group 1 (CMS) consisted of (a) one FB block with two extraoral implants and 10 pairs of magnetic abutments and (b) one P block with two metal housings and 10 pairs of magnetic attachments.
- Group 2 (NMS1) consisted of (a) one FB block with two extraoral implants and 10 pairs of NMS1 abutments and (b) one P block with two holes and 10 pairs of silicone attachments.
- Group 3 (NMS2) consisted of (a) one FB block with two extraoral implants and 10 pairs of NMS2 abutments and (b) one P block with two holes and 10 pairs of silicone attachments.

### 2.5. Measurement of the retentive forces

Cycles were established to evaluate the retention forces of the attachments. Considering the expectation that patients will use their prostheses for at least one year, the evaluation cycle was planned based on that duration. In general, patients with facial prostheses insert or remove their prostheses at least four times a day [21,22]. Accordingly, the maximum dislodging force for the attachments was measured after 0 (beginning), 120 (1 month), 360 (3 months), 720 (6 months), and 1440 (1 year) cycles of insertion and removal. In this in vitro study, the abutments were tested in the horizontal plane and in a parallel configuration for each cycle.

### 2.6. Experimental setup and study protocol

The measurement setup was designed as a horizontally positioned mechatronic system. Using a linear actuator (CCREAMT12 CC1012-01), force was applied to the system along a single axis, in both opening and closing directions, to ensure the completion of the cycle. At the 0th, 120th, 360th, 720th, and 1440th cycle points, the force required to disengage the system was measured using a digital dynamometer (PCE-FM). During the measurements, the digital dynamometer was precisely aligned horizontally with the axis of the implant and the support to ensure accurate readings. To facilitate and stabilize the movement of the structure (comprising the implant and support along with the linear actuator) into a single axis, guide and fixture structures were produced using a fused deposition modeling 3D printer, operating within a tolerance range of ±0.1%. The samples were placed inside the testing system, and the retentive forces were measured for all samples after each dislodging cycle. All measurements were recorded by a single researcher accompanied by a mechanical engineer.

### 2.7. Statistical analysis

The retentive forces measured using the described setup were recorded for all samples across all cycles. The measurements led to the following comparisons:

- The retentive forces of the three groups (CMS, NMS1, and NMS2) were compared for each dislodging cycle (0th, 120th, 360th, 720th, and 1440th cycles).
- For each cycle, the retentive forces of the different abutments were grouped and compared against each other.
- Pairwise comparisons across all groups were conducted to identify specific differences.

The Shapiro–Wilk test was used to test the normality of the data, and the results showed the data were not normally distributed (p < 0.05). The Kruskal–Wallis test was executed using base R functions to compare the groups, and Dunn’s posthoc test was conducted using the dunn.test package. The Friedman test was used to compare the dislodging cycles for each group. The Benjamini–Hochberg adjusted Wilcoxon sign-rank test was used for pairwise comparisons. To further explore the differences within each group across different cycles, the Friedman test was applied. This test was used to determine if there were statistically significant differences in retentive forces across the cycles within each group. Given the significant results from the Friedman test, pairwise Wilcoxon signed-rank tests were subsequently performed to identify which specific cycles within each group differed significantly. The p values from these pairwise comparisons were adjusted using the Benjamini–Hochberg method to control for false discovery rate. All statistical analyses were performed using R v4.3.1.

## 3. Results

This study compared the retentive forces of the CMS used for facial prostheses to those of two novel nonmagnetic abutment systems, NMS1 and NMS2. For each of the three experimental groups, the retentive forces were determined at the 0th, 120th, 360th, 720th, and 1440th dislodging cycles.

### 3.1. Retentive forces of the groups

The means and standard deviations of the retentive forces for the three groups at each dislodging cycle are shown in Table. Statistically significant differences were found between NMS1 and CMS and between NMS2 and CMS for each dislodging cycle (p < 0.01 for each). The retention forces of the CMS group were lower compared to the other two groups. Furthermore, for each dislodging cycle (0th, 120th, 360th, 720th, 1440th), the retention forces of NMS1 and NMS2 were similar (p = 0.413, p = 0.423, p = 0.389, p = 0.298, p = 0.332, respectively).

There were statistically significant differences in retention force values across the dislodging cycles for all three groups (p < 0.001 for each).

For the CMS group, the retention forces at the 0th and 120th dislodging cycles were found to be similar (p = 0.710). Moreover, it was observed that the retention forces decreased as the number of dislodging cycles increased.

**Figure 6:**
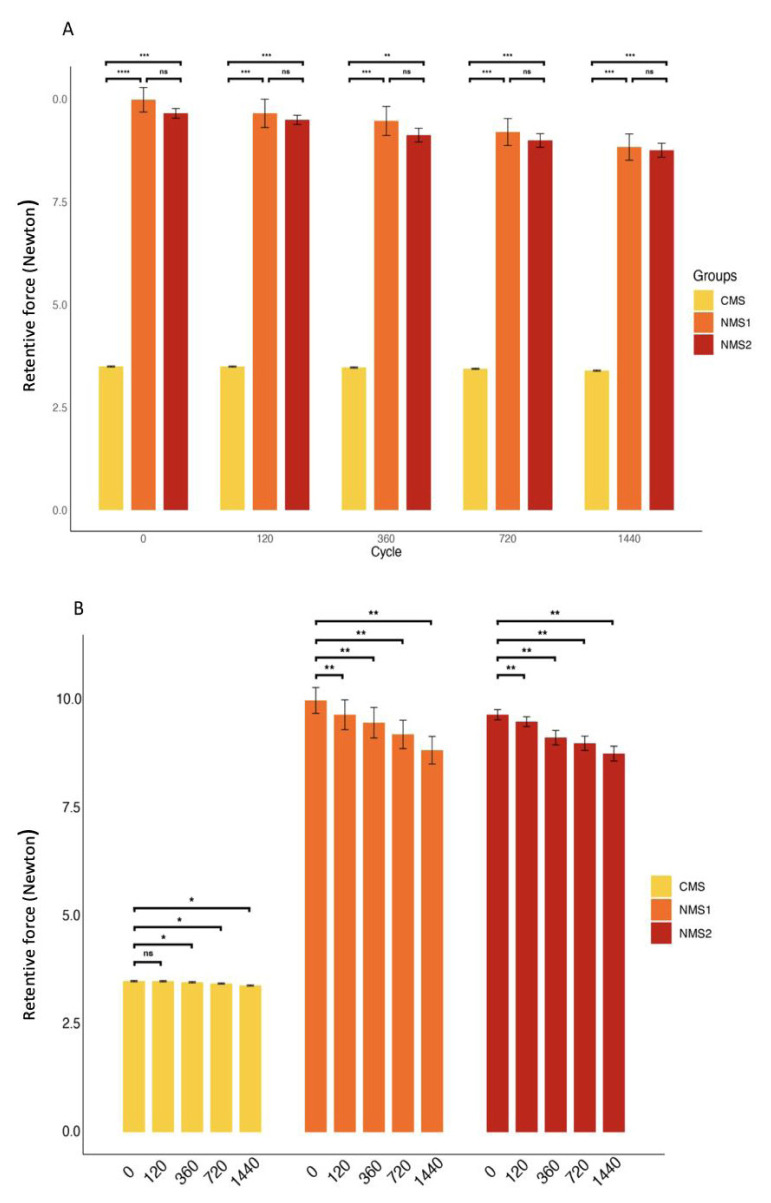
Progression of the mean retentive forces according to the different cycle tests. (A) Differences among the abutment types. (B) Differences across the cycles within each abutment type. Error bars represent the standard deviation of the mean. CMS = conventional magnetic system, NMS1 = nonmagnetic abutment system type 1, and NMS2 = nonmagnetic abutment system type 2. (ns: nonsignificant, * = p < 0.05, ** = p < 0.01, *** = p < 0.001, **** = p < 0.0001.)

For the NMS1 and NMS2 groups, statistically significant differences were found between all dislodging cycles, and it was observed that the retention forces decreased as the number of dislodging cycles increased. The progression of the mean retentive forces of the three groups is shown in Figure 6.

## 4. Discussion

In extraoral prostheses, retaining systems are one of the most critical components and the primary element responsible for the retention of the prosthesis. While there are many different designs, magnetic attachment systems have recently gained prominence. However, these systems also present certain disadvantages alongside their advantages. In this in vitro study, the retention forces of specially designed nonmagnetic attachment systems were compared with those of conventional magnetic abutments, and significant differences were observed between the systems. It was found that the retention forces decreased over time in all systems. Therefore, the null hypothesis, which argued that there were no significant differences between the nonmagnetic systems (NMS1 and NMS2) and the conventional magnetic system (CMS), was rejected.

Up to now, the basic retaining systems used for implant-supported facial prostheses have been ball/o-ring, bar-clip, and magnet systems [10–12]. Nowadays, materials such as polyetheretherketone (PEEK), which are more biocompatible, have a lower risk of tissue reaction, and possess chemical resistance, are preferred over older systems [23,24]. Although the bar-clip system may still be used, it has several limitations, such as fractures of the bars, the need for clip activation, loosening of bar screws and abutments, substructure fractures, loss of attachment between the silicone and the substructure, difficulty in maintaining hygiene, and the necessity of manual dexterity to replace the prostheses [20]. Therefore, the use of magnetic attachment systems has become more widespread.

Magnetic attachment systems have become more commonly used because they offer several advantages compared to other systems, including the ability to automatically position themselves when close to the correct position, a feature particularly useful for patients with limited manual dexterity [25]. In addition to facilitating prosthesis hygiene maintenance, magnets also help reduce the force exerted on implants and abutments during insertion and removal, which is a very straightforward process [18,26–28]. However, magnetic systems present challenges during routine patient follow-ups, as conventional magnetic abutments are not compatible with radiological imaging modalities, such as MRI. Several investigators have indicated that magnetic materials may pose potential dangers to MRI scanners and may cause artifacts in the scans [29–31]. Additionally, the procedures for removing and reinserting these components during repeated imaging can necessitate additional sessions [15,18,32].

In the present study, two novel nonmagnetic systems were developed for facial prostheses. The nonmagnetic abutments were designed with the expectation of achieving high retention abilities while causing minimal damage to the silicone material. For this purpose, two different designs were created. NMS1 has a more angular form, and NMS2 has a more rounded form. Titanium alloy was chosen for the production of both designs. Titanium is highly biocompatible, does not create artifacts in imaging, and does not need to be removed for imaging procedures [33,34]. Additionally, it contributes to cost efficiency, in contrast to magnets, which are more expensive due to their reliance on rare, costly materials and complex manufacturing processes.

The newly developed nonmagnetic systems in this study have the additional advantage of not requiring an attachment part to be inserted within the facial prosthesis. While magnetic systems provide retention through the use of both an abutment and an attachment, the nonmagnetic abutment design differs in how it provides retention; instead of automatic placement, undercut designs are used to achieve secure retention [27].

In their study comparing magnetic devices, Voigt et al. [35] examined the CMS system used in this study and reported it to have the lowest withdrawal strength. Additionally, consistent with our findings, they noted that as the head diameter increases, retention also increases. Although there is no established minimum retention force for extraoral prostheses, it has been reported to be between 5 and 7 N for overdenture prostheses [36]. In another study, the initial dislodging force of ball attachments with a similar interimplant distance was found to be 15.6 N, which is higher than in the present study [21]. While the retention force values in the current study were lower, they were still above the minimum retention force threshold. This difference is thought to be due to variations in head design.

Kobayashi et al. [37] demonstrated that retentive ball anchor attachments have high retention capacity and resistance to wear. However, it was observed that retentive forces decreased after several cycles. Other studies also support this finding [21,22]. Similarly, in the present study, the initial retentive forces were the highest, with a decrease observed after subsequent cycles. Although the change in retention force in the NMS systems was greater than in the CMS, they still provided 2.5 times more retention. Supporting our study, it has been observed that bar-clip attachments provide better retention than magnetic systems and exhibit higher durability, despite a decrease in retention over a three-year experimental period [38].

The present study evaluated retentive force, an important parameter in facial prostheses, but it also has several limitations. The first limitation is that the study was conducted under in vitro conditions, which may not fully replicate the behavior of live tissues. Therefore, it is advisable not to directly translate the results to in vivo outcomes. Clinically, there is also the possibility that components may not always be placed parallel, and interimplant distances may vary. Future studies could evaluate magnetic and nonmagnetic systems in different configurations. This would aid in producing facial prostheses that provide optimal comfort for patients. Although the system used in the present study is made from biocompatible materials and is suitable for imaging, clinical studies are still necessary.

## 5. Conclusion

The novel nonmagnetic systems evaluated in this study demonstrated higher retention forces compared to magnetic systems, indicating that these systems could be a reliable alternative for facial prostheses. Across all systems, a decrease in retention forces was observed with the increasing number of insertion and removal cycles over time. This factor is important to consider for the long-term use of facial prostheses.

## Figures and Tables

**Table:** Retentive forces of the three abutment types at each dislodging cycle.

	Retentive forces (Newtons)			p*
Dislodging cycles	CMS n =10 Mean ± SD	NMS1 n = 10 Mean ± SD	NMS2 n = 10 Mean ± SD	
0	3.49 ± 0.04^aA^	9.98 ± 0.89^bA^	9.65 ± 0.35^bA^	<0.01
120	3.49 ± 0.04^aA^	9.65 ± 1.03^bB^	9.49 ± 0.33^bB^	<0.01
360	3.46 ± 0.04^aB^	9.47 ± 1.05^bC^	9.12 ± 0.50^bC^	<0.01
720	3.43 ± 0.03^aC^	9.20 ± 0.98^bD^	8.99 ± 0.49^bD^	<0.01
1440	3.39 ± 0.04^aD^	8.83 ± 0.95^bE^	8.75 ± 0.51^bE^	<0.01
p**	<0.001	<0.001	<0.001	

CMS = conventional magnetic system, NMS1 = nonmagnetic abutment system type 1, and NMS2 = nonmagnetic abutment system type 2.

* = Kruskal–Wallis H test and

** = Friedman test was conducted.

Different lowercase letters in rows indicate a statistically significant difference for different abutment types (p < 0.05). Different uppercase letters in columns indicate a statistically significant difference for different cycles (p < 0.05).

## Data Availability

Data and materials are available upon reasonable request.
